# Complete Genome Sequence of the Highly Adhesive Bacterium Acinetobacter sp. Strain Tol 5

**DOI:** 10.1128/MRA.00567-21

**Published:** 2021-09-02

**Authors:** Masahito Ishikawa, Katsutoshi Hori

**Affiliations:** a Department of Biomolecular Engineering, Graduate School of Engineering, Nagoya University, Nagoya, Aichi, Japan; b PRESTO, Japan Science and Technology Agency, Kawaguchi, Saitama, Japan; Loyola University Chicago

## Abstract

Acinetobacter sp. strain Tol 5 is a nonpathogenic Gram-negative bacterium with biotechnological and environmental applications. Here, we report the complete genome sequence of Acinetobacter sp. strain Tol 5, which has a genome size of 4,799,506 bp and a G+C content of 38.1%.

## ANNOUNCEMENT

Acinetobacter sp. strain Tol 5 was isolated as a toluene-degrading bacterium from a biofiltration unit used for treating off-gas containing toluene (1). Tol 5 can metabolize various chemicals, including toluene (2). Tol 5 exhibits nonspecific and high adhesiveness to various material surfaces through the adhesive bacterionanofiber protein AtaA, which is localized on the cell surface (3, 4). These metabolic and adhesive traits of Tol 5 are beneficial for biotechnological and environmental applications (1, 5, 6). Here, we report the complete genome sequence of Tol 5.

Tol 5 was inoculated into 2 ml of Luria-Bertani (LB) medium in a conical tube and incubated at 28°C with shaking. Two hundred microliters of the overnight culture was added to 20 ml of fresh LB medium in a flask and incubated at 28°C until the optical density at 660 nm reached 0.5. Then, 200 μl of 180 μg/ml chloramphenicol was added to the culture, followed by further incubation at the same temperature for 1 h. After Tol 5 cells were harvested via centrifugation, genomic DNA was extracted using a Genomic-tip 500/G kit (Qiagen, USA). The extracted DNA was analyzed using a MinION sequencer (Oxford Nanopore Technologies [ONT], UK) and using an iSeq 100 sequencer (Illumina, USA). A sequencing library for MinION sequencing was prepared using a rapid sequencing kit (SQK-RAD004; ONT). MinION sequencing was performed using R9.4.1 flow cells (FLO-MIN106; ONT). In contrast, a sequencing library for iSeq 100 sequencing was prepared using the Nextera DNA Flex library preparation kit (Illumina) and Nextera DNA CD indexes (Illumina). A total of 404,000 reads (*N*_50_, 13,214 bp; maximum length, 100,337 bp; coverage, 455×) and 446,673 reads (2 × 150-bp paired-end reads; coverage, 27×) were obtained from the MinION and iSeq 100 sequencing, respectively. Default parameters were used to run the following software unless otherwise indicated. MINKNOW software v3.1.20 (ONT) was used for data acquisition from the MinION sequencer. Real-time base calling was performed with MinIT (ONT) and integrated Guppy v2.0.10 software (ONT) to generate FAST5 and FASTQ files. The MinION reads were filtered by quality and size (scores of >Q10 and size of 15,000 bp) using NanoFilt v2.5.0 (7), whereas the iSeq 100 reads were filtered by quality (scores of >Q20), and 1 bp was trimmed from the 3′ end using Trim Galore! v0.6.0 (https://www.bioinformatics.babraham.ac.uk/projects/trim_galore).

Hybrid *de novo* assembly was performed using Unicycler v0.4.4 (8), revealing two circular contigs; the chromosome was 4,681,789 bp, with a G+C content of 38.0%, and the plasmid was 117,717 bp, with a G+C content of 40.8%. The genome of Tol 5 was automatically annotated using DFAST v1.2.13 (9). Some of the annotations were revised manually based on BLASTP search results. We identified 4,402 coding sequences, including 81 tRNAs, and 21 rRNAs. We expect that the complete genome sequence reported here will facilitate the use of Acinetobacter strains for environmental and biotechnological applications.

### Data availability.

The genome sequence reported here was deposited in DDBJ/GenBank under the accession numbers AP024708 and AP024709. The raw reads were deposited into the Sequence Read Achieve (SRA) database with accession numbers DRR295876 and DRR295877. The associated BioProject and BioSample accession numbers are PRJDB11707 and SAMD00324391, respectively.
